# Bone marrow-derived stem cells ameliorate hepatic fibrosis by down-regulating interleukin-17

**DOI:** 10.1186/2045-3701-3-46

**Published:** 2013-12-06

**Authors:** Linhua Zheng, Jindong Chu, Yongquan Shi, Xinmin Zhou, Ling Tan, Qiang Li, Lina Cui, Zheyi Han, Ying Han, Daiming Fan

**Affiliations:** 1 State Key Laboratory of Cancer Biology & Xijing Hospital of Digestive Disease, Xijing Hospital, Fourth Military Medical University, 127 Changle Western Road, Xi’an 710032, Shaanxi Province, China; 2 Department of Pediatric Dentistry, School of Stomatology, Fourth Military Medical University, Xi’an 710032, China

**Keywords:** Bone marrow-derived stem cells, Decompensated cirrhosis, IL-17, Immunoregulation, HBV, Carbon tetrachloride

## Abstract

**Background:**

Accumulating evidences have identified the immunoregulatory features of stem cells. In this study, the immunoregulation of bone marrow-derived stem cells (BMSCs) transplanted into patients with HBV-related decompensated cirrhosis and mouse model of liver injury induced by carbon tetrachloride (CCl_4_) administration was observed.

**Results:**

Compared with healthy controls, patients with HBV-related decompensated cirrhosis showed significantly higher levels of TNF-alpha, IL-12, TGF-beta1, IL-17, and IL-8. However, only IL-17 was markedly decreased after autologous BMSCs transplantation during their follow-up. The same results were found in the CCl_4_-treated mice. Furthermore, we found that exogenous IL-17 partly abolished the therapeutic effect of BMSCs whereas IL-17-specific antibody promoted improvement of liver injury in CCl_4_-treated mice, resembling the therapeutic effect of BMSCs transplantation.

**Conclusions:**

These data suggested that BMSCs transplantation induces a decrease of IL-17 level, which at least in part delineates the mechanisms of stem cells-mediated therapeutic benefit on liver disease.

## Background

More recently, advances in the understanding of bone marrow-derived stem cells (BMSCs) biology and plasticity have raised hopes that stem cell therapy may offer exciting therapeutic possibilities for patients with liver diseases [1,2]. However, there is much debate concerning the mechanisms by which BMSCs contribute to hepatic regeneration. Recently, more and more evidence have identified the strong immunoregulatory effect of stem cells [3,4].

It is well known that inflammation is a critical factor in the initiation and maintenance of liver fibrogenesis. When liver injuries occur, damaged epithelial and/or endothelial cells release inflammatory mediators that recruit peripheral blood inflammatory cells to the damaged liver and release of fibrosis-related mediators including TGF-beta1 and TNF-alpha, triggering activation of hepatic stellate cells and formation of extracellular matrix [5]. Recently, some reports suggest that proinflammatory cytokine IL-17 (also known as IL-17A) plays an important role in many liver diseases, including alcoholic liver disease, hepatocellular carcinoma, autoimmune liver disease, acute and chronic hepatitis B and it is associated with the disease progression [6-9]. Cytokines, signaling proteins produced by different kinds of cell types, are essential for the development and function of both innate and adaptive immune response. However, the regulatory effect of BMSCs on some kinds of cytokines is still obscure.

On the basis of previous studies, we initiated a clinical study analyzing the regulation of BMSCs on several kinds of cytokines after transplanting into HBV-related decompensated cirrhotic patients. And then we verified our clinical results using a well-estimated mouse model of carbon tetrachloride (CCl_4_)-induced liver injury and we firstly found that the therapeutic effect of BMSCs was, at least in part, due to their down-regulation of IL-17.

## Results

### Therapeutic effect of autologous BMSCs transplantation on patients with HBV-related decompensated cirrhosis

Data for serum albumin (ALB), prothrombin activity (PTA), and cholinesterase (CHE) were collected from the enrolled patients at baseline (1 week before transplantation), and at 4, 12, 24, 36 and 48 weeks after BMSCs transplantation, respectively. As shown in Table 1, compared with baseline, the average levels of serum ALB, PTA and CHE had markedly increased at 4 weeks after transplantation. And Child-Turcotte- Pugh (CTP) and Model for End-Stage Liver Disease (MELD) scores were gradually decreased and reached statistical difference at 4 weeks. No serious adverse effect was observed after transplantation.

**Table 1:** Improved liver function of patients after bone marrow-derived stem cells (BMSCs) transplantation

**Index**	**Weeks**					
	**Baseline**	**4w**	**12w**	**24w**	**36w**	**48w**
Albumin(g/L)	29.25(3.80)	34.83(4.87)*	35.98(4.36)*	40.03(5.42)*	38.11(5.28)*	39.27(6.65)*
PTA(%)	51.50(15.51)	61.46(17.80)*	64.23(13.45)*	72.17(14.05)*	69.40(11.37)*	62.91(13.42)*
CHE(U/L)	2766.43(913.79)	3527.94(1113.25)*	3625.55(1150.36)*	4168.91(1403.57)*	4438.27(1032.26)*	4902.87(1771.91)*
CTP score	9.05(1.94)	7.25(1.98)*	7.03(2.16)*	6.45(1.60)*	6.52(1.93)*	6.64(1.69)*
MELD score	14.04(4.89)	12.09(3.31)*	11.33(4.60)*	10.34(4.93)*	10.40(4.09)*	10.52(6.36)*

Values are expressed as the mean (standard deviation).

*p < 0.05 in comparison with baseline. Baseline, 1 week before transplantation.

PTA, prothrombin activity; CHE, cholinesterase; CTP, Child-Turcotte-Pugh; MELD, Model for End-Stage Liver Disease.

### Decrease of serum IL-17 after autologous BMSCs transplantation in patients

**Figure 1:**
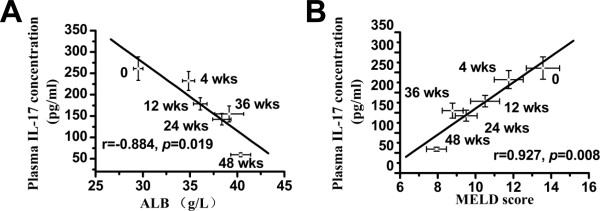
**Relationships between IL-17 level and liver function in patients.** The relationships between IL-17 level and ALB **(A)** and MELD score **(B)** were calculated using the Spearman rank correlation test. The solid line represents the linear growth trend, r means correlation coefficient. ALB, albumin; MELD score, Model for End-Stage Liver Disease score.

The results showed that serum TNF-alpha, IL-12, TGF-beta1, IL-17, and IL-8 in HBV-related decompensated cirrhotic patients were significantly higher than that in age- and sex-matched healthy controls. But the changes of IFN-gamma and IL-13 displayed no statistical significance (Table 2). IL-4 was below the assay detection limit and was not detectable both in patients and in healthy controls. Then we examined the changes of the aforementioned cytokines in patients after BMSCs transplantation during entire follow-up. As shown in Table 2, compared with baseline, only IL-17 was found to be significantly decreased at 24 weeks after BMSCs transplantation (*p* < 0.01), and kept on declining through the entire follow-up. Interestingly, there was a close relationship between serum IL-17 and liver function. A highly negative correlation was observed between serum IL-17 and ALB (r = −0.884, *p* = 0.019), but a positive correlation existed between serum IL-17 and MELD score (r = 0.927, *p* = 0.008) (Figure 1).

**Table 2:** Dynamic changes of cytokines after bone marrow-derived stem cells (BMSCs) transplantation

**Cytokines**	**HC**	**Weeks after BMSCs transplantation**					
		**Baseline**	**4w**	**12w**	**24w**	**36w**	**48w**
TNF-α	1.8(1.2)	36.2(7.6)^a^	34.1(12.3)	38.3(9.2)	32.4(5.3)	31.6(2.5)	32.5(5.7)
IFN-γ	3.0(1.1)	2.0(0.8)	4.5(1.3)	1.0(0.7)	1.7(0.8)	2.6(0.4)	3.7(1.1)
IL-12	3.2(0.6)	23.2(1.4)^a^	21.4(2.1)	18.9(1.7)	20.1(2.2)	19.6(1.5)	20.5(1.2)
IL-13	5.6(1.4)	5.7(2.0)	6.1(1.2)	7.5(2.1)	6.0(1.5)	5.8(1.2)	5.7(1.5)
TGF-β1	8.9(1.3)	23.4(8.3)^a^	26.5(7.6)	18.2(6.2)	21.6(3.7)	19.5(9.1)	22.2(4.3)
IL-17	21.2(18.9)	216.4(109.3)^a^	269.8(112.4)	189.0(108.2)	106.1(92.3)^b^	118.2(102.9)^b^	57.0(32.7)^b^
IL-8	17.2(10.3)	131.4(59.3)^a^	142.9(71.5)	129.3(50.2)	150.2(48.2)	136.5(32.7)	132.4(70.2)

Values are expressed as the mean (standard deviation).

^a^*p*<0.05 in comparison with HC; ^b^*p*<0.05 in comparison with Baseline.

HC, healthy controls; Baseline, 1 week before transplantation.

### Change of IL-17 in mouse model of liver injury induced by CCl_4_ injection

**Figure 2:**
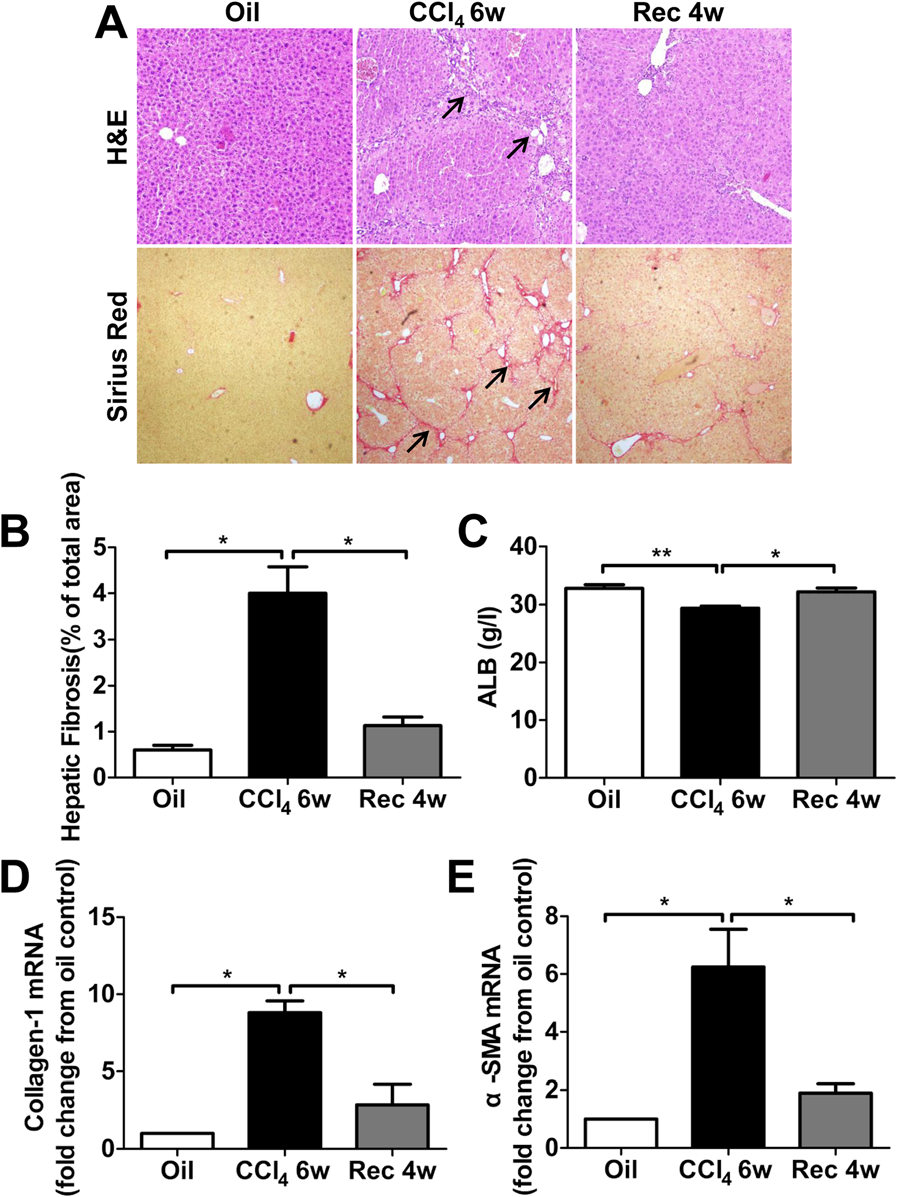
**Liver injury induced by CCl**_**4 **_**administration.** The mice were repeatedly treated with oil alone or CCl_4_ mixture for 6 weeks to induce liver injury, and then stopped injection to observe spontaneous recovery for 4 weeks. **(A)** Liver inflammatory (upper panels arrowhead) and hepatic collagen deposition (lower panels arrowhead) were determined by H&E and Sirius red staining. Original magnifications: upper panels × 100, lower panels × 40. **(B)** The percentage of stained red area of Sirius red was assessed at 100 × magnification with a quantitative image analyzer. **(C)** Serum chemistry of liver function was performed for albumin (ALB). The expressions of alpha- smooth muscle actin (SMA) **(D)** and collagen-1 **(E)** mRNA in liver tissues were measured by real time (RT)-PCR. CCl_4_ 6w, 6-week CCl_4_ injection; Rec 4w, stopped CCl_4_ injection for 4-week to recover spontaneously. *p < 0.05, **p < 0.01.

CCl_4_ injection to mice for 6 weeks resulted in overt but reversible liver injury. As shown in Figure 2, after repeatedly 6-week CCl_4_ injection, when compared with oil-treated controls, hematoxylin and eosin (H&E) and Sirius red staining showed a visible inflammation and fibrosis. And serum ALB declined statistically. The expressions of alpha- smooth muscle actin (SMA) and collagen-1 mRNA in liver tissues measured by real time-polymerase chain reaction (RT-PCR) significantly increased. However, the aforementioned changes were gradually reversed to nearly normal levels after 4 weeks stopped CCl_4_ injection (Figure 2).

**Figure 3:**
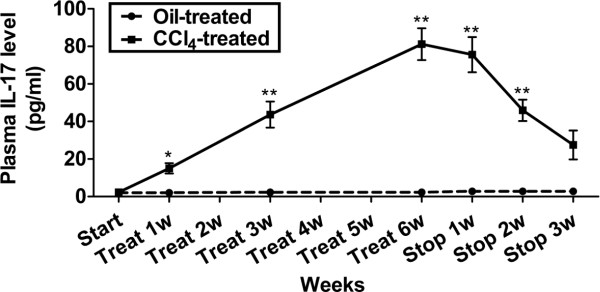
**Change of IL-17 in the pathogenesis of mouse liver injury induced by CCl**_**4 **_**injection.** Solid line and dash line showing the dynamic change of plasma IL-17 in the pathogenesis of mouse liver injury induced by CCl_4_ injection. *p < 0.05, **p < 0.01 *vs* oil-treated group. Start, the time when oil or CCl_4_ injection started; Treat, oil or CCl_4_ treatment; Stop, stopped oil or CCl_4_ injection.

Then we measured the dynamic changes of IL-17 in the pathogenesis of mouse liver injury induced by CCl_4_ injection. Statistically, serum IL-17 level increased significantly during 6-week CCl_4_ administration, but then gradually decreased after 4 weeks of stopped CCl_4_ injection compared with oil treated control (Figure 3). This indicates a closed relationship between IL-17 level and severity of liver injury.

### A causative role of IL-17 in the pathogenesis of liver injury in mice

**Figure 4:**
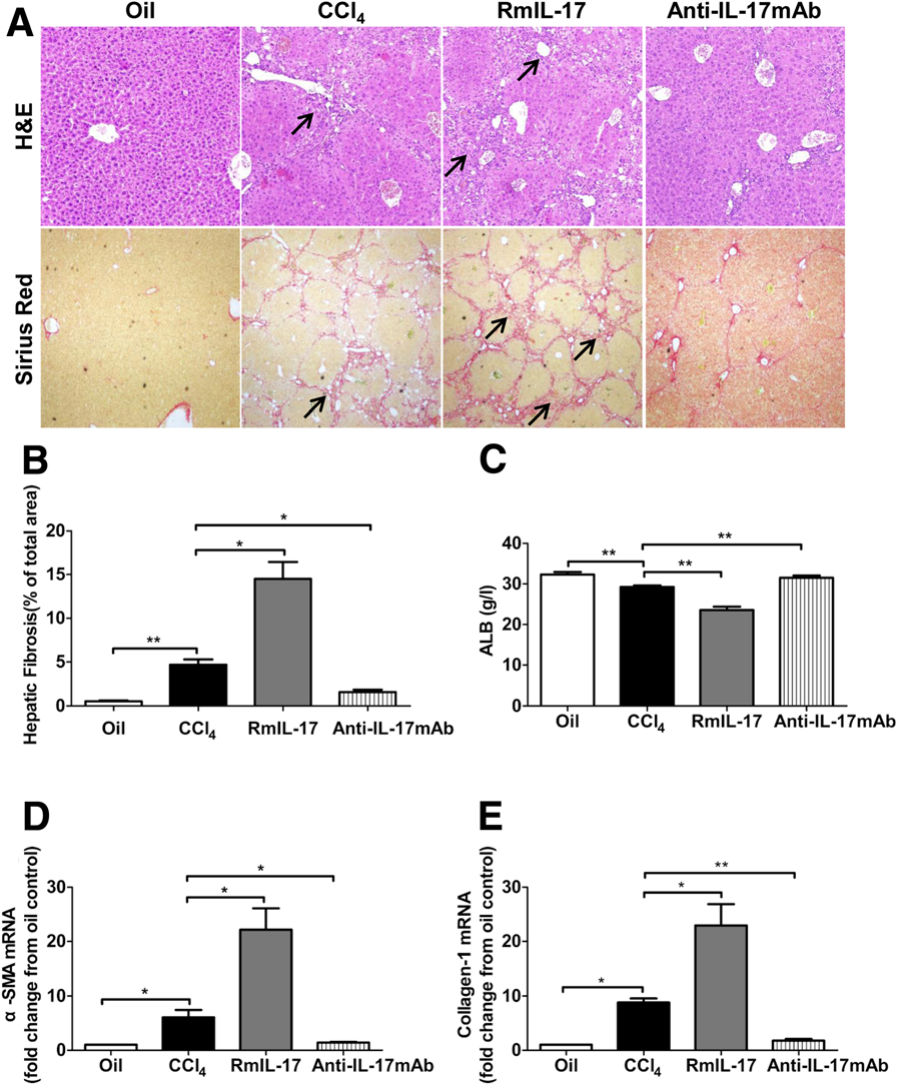
**Key role of IL-17 in the pathogenesis of liver injury induced by CCl**_**4 **_**injection.** Before CCl_4_ injection, mice were given monoclonal rat anti-mouse IL-17 antibody (anti-mouse IL-17 mAb) or recombinant mouse (rm) IL-17. **(A)** Liver inflammatory (upper panels arrowhead) and hepatic collagen deposition (lower panels arrowhead) were determined by H&E and Sirius red staining. Original magnifications: upper panels × 100, lower panels × 40. **(B)** The percentage of stained red area of Sirius red was assessed at 100 × magnification with a quantitative image analyzer. **(C)** Serum chemistry of liver function was performed for albumin (ALB). The expressions of alpha- smooth muscle actin (SMA) **(D)** and collagen-1 **(E)** mRNA in liver tissues were measured by real time (RT)-PCR. *p < 0.05, **p < 0.01.

To investigate whether IL-17 has a causative effect in the CCl_4_-induced liver injury, we changed IL-17 level *in vivo* by administration of exogenous recombinant mouse (rm) IL-17 or blockade of endogenous IL-17 with neutralizing IL-17-specific antibody (anti-IL-17 mAb). H&E and Sirius red staining showed that liver inflammation and fibrosis were significantly exacerbated by injection of rmIL-17, but ameliorated by neutralizing anti-IL-17 mAb compared with only CCl_4_ injection group (Figure 4A, B). The serum ALB in only CCl_4_ group was higher than that in rmIL-17 group, but significantly lower than that in anti-IL-17 mAb group (Figure 4C). Similarly, RT-PCR analysis confirmed that the hepatic mRNA expressions of alpha-SMA and collagen-1 were significantly increased in rmIL-17 injected group, but markedly decreased in anti-IL-17mAb treated mice compared with controls (Figure 4D, E). IL-17 levels were significantly increased in rmIL-17-injected group, but decreased in anti-IL-17 mAb-treated group compared with control groups (data not shown).

### BMSCs transplantation ameliorates CCl_4_-induced liver injury and down-regulates IL-17 level in mice

**Figure 5:**
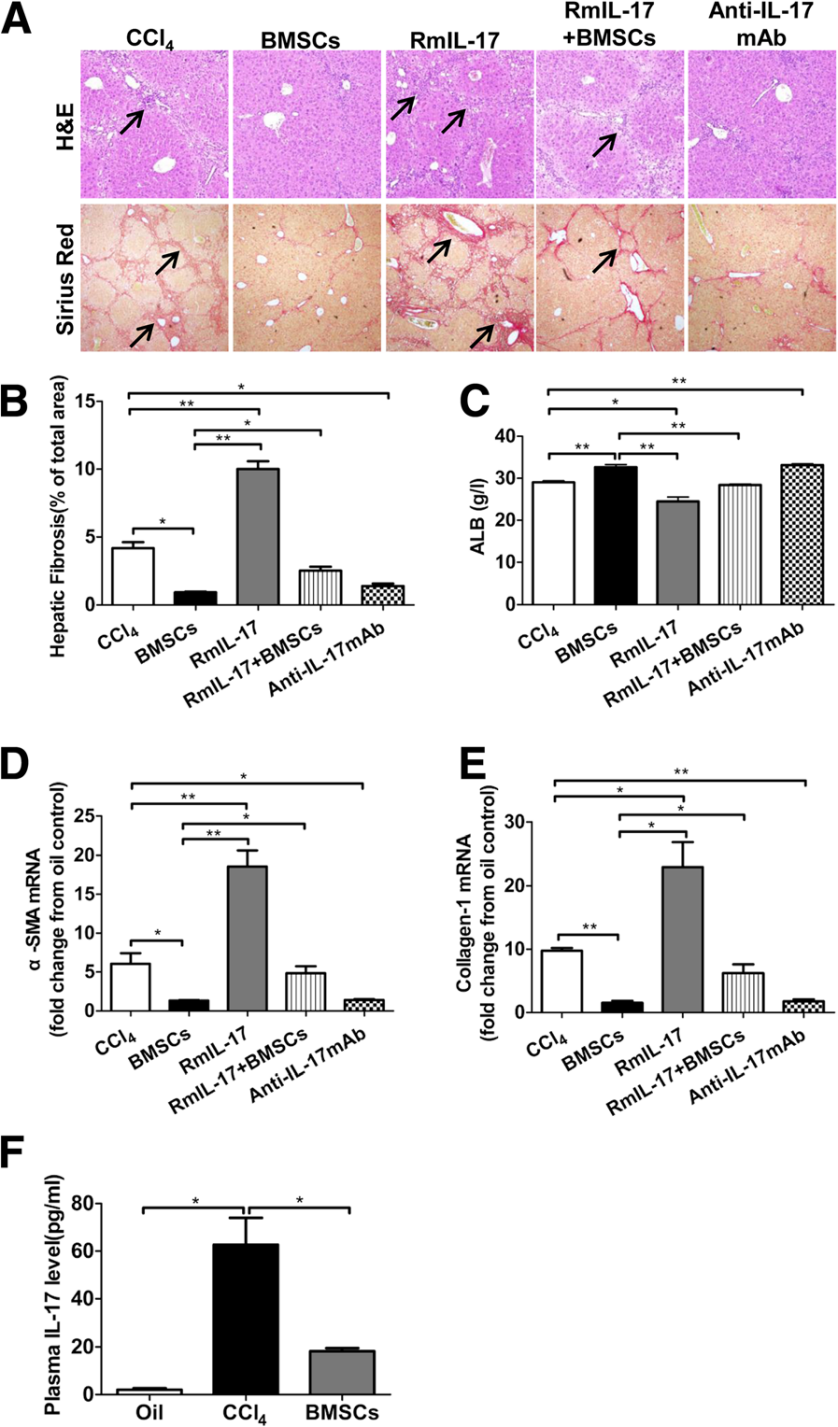
**IL-17 is a key for BMSCs-mediated amelioration of liver injury induced by CCl**_**4 **_**injection.** After 6 weeks of CCl_4_ administration to induced liver injury, the mice were randomly divided into several groups and were given a tail vein injection of BMSCs (5 × 10^6^ cells in 0.2 ml PBS), a intraperitoneal injection of rmIL-17, a intraperitoneal injection of rmIL-17 and a tail vein injection of BMSCs, and five intraperitoneal injections of anti-mouse IL-17 mAb, respectively. Then the mice continued to be given CCl_4_ injection during our study to maintain liver fibrotic status. One group mice were only given CCl_4_ injection during the whole study and served as control. **(A)** Liver inflammatory (upper panels arrowhead) and hepatic collagen deposition (lower panels arrowhead) were determined by H&E and Sirius red staining. Original magnifications: left panels × 100, right panels × 40. **(B)** The percentage of stained red area of Sirius red was assessed at 100 × magnification with a quantitative image analyzer. **(C)** Serum chemistry of liver function was performed for albumin (ALB). The expressions of alpha- smooth muscle actin (SMA) **(D)** and collagen-1 **(E)** mRNA in liver tissues were measured by real time (RT)-PCR. **(F)** The plasma IL-17 level was analyzed by enzyme-linked immunosorbent assay (ELISA). Results represent means and standard deviation (SD) of at least five mice per group. BMSCs, bone marrow-derived stem cells. *p < 0.05, **p < 0.01.

Transplantation of BMSCs to CCl_4_-treated mice significantly improved not only liver function, but also liver fibrosis. As shown in Figure 5A and B, H&E and Sirius red staining showed that liver inflammation and fibrosis were significantly ameliorated after BMSCs transplantation. Compared with CCl_4_-treated control, serum ALB in BMSCs-transplanted mice increased statistically, and the expressions of alpha-SMA and collagen-1 mRNA in liver tissues significantly declined (Figure 5C-E). Strikingly, serum IL-17 decreased markedly after BMSCs transplantation compared control (Figure 5F).

### IL-17 is a key for BMSCs-mediated amelioration of liver injury in mice

In an attempt to determine whether decrease of IL-17 levels could be linked to the therapeutic benefits of BMSCs transplantation on CCl_4_-induced liver injury, the 6-week CCl_4_-treate mice were randomly divided into several groups as described in the Material and Methods part. As shown in Figure 5, H&E and Sirius red staining showed that the areas of liver fibrosis in BMSCs and anti-IL-17 mAb groups were significant decreased than that in only CCl_4_-treated group. However, there was no significant difference between BMSCs and Anti-IL-17 mAb groups. Meanwhile, the area of liver fibrosis in rmIL-17 group was markedly increased than that in only CCl_4_-treated or BMSCs groups. Strikingly, the difference in the area of liver fibrosis between BMSCs and rmIL-17 + BMSCs groups was overt (*p* < 0.05). These results were confirmed by the hepatic mRNA expressions of alpha-SMA and collagen-1 in liver tissues. Moreover, serum ALB in BMSCs and anti-IL-17 mAb groups was significant higher than that in only CCl_4_-treated group. And there was no significant difference between BMSCs and Anti-IL-17 mAb groups. Serum ALB in rmIL-17 + BMSCs group displayed significantly lower than that in BMSCs group, indicating exogenous IL-17 could block the therapeutic effect of BMSCs.

## Discussion

More recently, the therapeutic effect of stem cell transplantation on liver diseases have been investigated in mice and human, yet the underlying mechanisms are obscure. The present study demonstrates that transplantation of autologous BMSCs could down-regulate serum IL-17 in patients with HBV-related decompensated cirrhosis and in mouse model of CCl_4_-induced liver injury, which may at least partly delineate the mechanisms of BMSCs-mediated therapeutic benefit on liver diseases.

The stem cells mostly used to transplant are derived from bone marrow including mesenchymal stem cells (MSCs), hematopoietic stem cells (HSCs) and unsorted mononuclear cells. However, which cell type is more effective in treating liver diseases is largely unknown. Recently, several studies have suggested that MSCs and HSCs function synergistically for the therapy of diabetes and heart failure [10,11]. The rationale for the synergistic actions of MSCs and HSCs may be that MSCs provide a microenvironment for HSCs in both embryonic and postnatal stage [12]. Thus, the mixture of bone marrow stem cells was used in our study.

Consistent with previous reports [2,13,14], we demonstrated that most HBV-related decompensated cirrhotic patients with BMSCs transplantation displayed significantly improved liver function during 48 weeks of follow-up. As shown in Table 1, signs of clinical improvement of liver function, ALB, PTA, CHE, CTP and MELD scores, were observed after transplantation. And the peak of improvement was at 24 weeks after transplantation. Considering the high risk of liver biopsy in end-stage liver disease patients, we did not perform histological analysis. These data suggest that BMSCs transplantation, at least in short term, may be an effective and safe therapeutic approach for HBV-related decompensated cirrhosis.

With accumulating evidences about the implication of inflammation in the pathogenesis of diseases, the immunoregulatory features of stem cells have drawn more attention. It has been reported that MSCs could inhibit the function of T-lymphocytes, natural killer (NK) cells and dendritic cells, and regulate their cytokine spectrums, showing an immunoregulatory capacity *in vitro* and *in vivo*[15-17]. Recently, Suh *et al.*[18] investigated that bone marrow cells have the capacity of anti-inflammation and anti-fibrosis through expression of IL-10. And some studies have reported that MSCs can prevent naive CD4^+^ T cells from differentiating into Th17 cells and inhibit the production of IL-17 *in vitro*[19,20]. However, some other studies indicate that co-culture of splenocytes and MSCs *in vitro* could increase IL-17 secretion [21,22]. The opposite regulatory effect of MSCs on the production of IL-17 may be related to the different co-culture systems *in vitro*. *In vivo* study, we found that serum IL-17 in patients with HBV-related decompensated cirrhosis statistically decreased at 24 weeks after BMSCs transplantation when compared with baseline. It is noteworthy that there was a highly negative correlation between serum IL-17 and ALB, but a positive correlation between serum IL-17 and MELD score. However, the other cytokines, aforementioned, were not significantly changed after transplantation. And in the mouse model of CCl_4_-induced liver injury, IL-17 level also markedly declined after transplantation of homologous BMSCs.

IL-17, produced by CD4^+^ Th17 cells, NKT cells, mast cells, neutrophils and gamma delta T cells [23,24], plays a major role in host protection against extracellular pathogens and induction of tissue inflammation [25]. It has been found that IL-17 plays a critical role in pulmonary fibrosis [26] and psoriasis [27]. And some recent clinical studies have been reported that utilization of human anti-IL-17 monoclonal antibody [28] or human anti-IL-17-receptor monoclonal antibody [29] could improve clinical symptoms of psoriasis. And emerging evidences have indicated that IL-17 is implicated in the induction of liver injury and liver fibrosis both in human and in mice [30,31]. Similarly, we found that not only HBV-related decompensated cirrhotic patients, but also mouse model of liver injury induced by CCl_4_ administration displayed significant higher serum IL-17 levels than that in healthy controls. And in mice, a dynamic change of IL-17 level was observed during the progression of well-established CCl_4_-induced liver injury in mice. Moreover, the deteriorated effect of exogenous IL-17 and protective effect of neutralizing anti-IL-17 antibody were apparent during the procession of liver injury. Recent study found that IL-17 induces liver injury and fibrosis may through two mechanisms: IL-17 stimulates kupffer cells to express other inflammatory cytokine, such as TNF-alpha, and fibrogenic cytokine TGF-beta1; IL-17 directly stimulates hepatic stellate cells to express collagen and promotes their activation into fibrogenic myofibroblast via Stat3 [30]. These results suggest that IL-17 has a strong profibrogenic effect in the pathogenesis of liver disease and it is positively correlated with the severity of liver injury. However, which cell type is the main producer of IL-17 in the pathogenesis of liver injury, and how IL-17 induces liver injury and fibrosis need to be further pursued.

However, the question in our results is that we cannot distinguish cause and effect. Was the down-regulation of serum IL-17 a cause of the therapeutic effect of BMSCs on patients/mice with liver diseases, or was merely a satellite phenomenon? In the mouse model of CCl_4_-induced liver injury, we found that blockage of endogenous IL-17 with anti-IL-17-specific antibody could promote improvement of liver injury, resembling the therapeutic effect of BMSCs transplantation. It suggests that amelioration of liver injury may be due to the down-regulation of IL-17 either by neutralizing anti-IL-17 antibody or by BMSCs transplantation. Furthermore, injection of exogenous rmIL-17 significantly exacerbated liver injury, and even abolished the therapeutic effect of BMSCs transplantation on CCl_4_-induced mouse liver injury, indicating down-regulating IL-17 is a key for BMSCs-mediated amelioration of hepatic injury in mice. However, how BMSCs regulates IL-17 will be our next studies.

It should admit the shortcomings in our study that the mouse model of CCl_4_-induced liver injury, which has long been used as a valuable model to investigate the pathophysiological mechanism of liver injury and fibrosis, is different from patients with HBV-related decompensated cirrhosis. Therefore, it will be helpful in the future to utilize two or more animal models to clarify the role of IL-17 in the process of BMSCs-mediated amelioration of liver diseases.

## Conclusions

Here, we have shown that IL-17 increases significantly in HBV-related decompensated cirrhotic patients and CCl_4_-treated mice compare with controls. And a highly negative correlation between IL-17 and liver function indeed exists. Moreover, the deteriorated effect of exogenous IL-17 and protective effect of neutralizing anti-IL-17 antibody are apparent during the procession of CCl_4_-induced liver injury in mice.

A marked decrease of IL-17 after BMSCs transplantation in patients and CCl_4_-treated mice has been found. In animal study, it is demonstrated that exogenous IL-17 partly abolishes the therapeutic effect of BMSCs whereas IL-17-specific antibody promotes improvement of liver injury in CCl_4_-treated mice, resembling the therapeutic effect of BMSCs transplantation.

Overall, we reveal a previously unrecognized finding that BMSCs transplantation exerts their beneficial action on liver diseases, at least partly, through down-regulating IL-17.

## Material and methods

### Human studies

#### Patients

All 42 HBV-related decompensated cirrhotic patients received mobilized BMSCs transplantation were hospitalized in Xijing Hospital from July 2009 to December 2010. Patients were included if they were 18–70 years of age, with clinical, biochemical, sonograhpic, and histology diagnosis of HBV-related decompensated liver cirrhosis. Evidences of decompensated liver cirrhosis were at screening, such as portal hypertension, serum ALB, bilirubin, PTA, abdominal ultrasonography and CTP score, and alpha-fetoprotein and liver imaging were detected. Exclusion criteria were presence of active moderate to severe hepatic encephalopathy, spontaneous peritonitis or variceal bleeding during 1 mouth before enrollment; refractory ascites; hemoglobin ≤ 7.0 g/L, platelet <300 000/ul; serum Cr>2 mg/dL at screening; positive HIV or HCV antibody; active thrombosis of the portal, hepatic or splenic veins; hepatocellular carcinoma or other malignancies; active infectious disease; evidences of active autoimmune liver disease (e.g. gamma globulin > twice normal, ALT > 3 times normal in patients with autoimmune hepatitis); lines of evidence of extrahepatic biliary disease (e.g. presence of primary sclerosing cholangitis or bile duct stone); presence of severe comorbid diseases (e.g. severe renal, respiratory, or cardiac disease); history of alcohol or hepatotoxic drugs use within the last 6 months before enrollment; unwilling to assign the informed consent; death or missing before endpoint of follow-up. All the patients enrolled first received 1 week of routing medical treatment (baseline time point), and then received the transplantation of autologous BMSCs. The protocol of mobilized BMSCs transplantation was described in our previous study [32]. Patients received recombinant granulocyte-colony stimulating factor (G-CSF) (Qi Lu Pharmaceutical Co. Ltd, Jinan, China) subcutaneously at 5–10 μg/kg/day for 4 days to induce BMSCs into peripheral blood. BMSCs were collected by means of apheresis using the COBER Spectra™ Apheresis System (Gambro BCT Inc., Stockholm, Sweden). When the number of BMSCs reached to 10^7^–10^8^/kg, 60 ml BMSCs were returned to the patient via the hepatic artery in the imaging department of our hospital. During our follow-up, enrolled patients received regular anti-viral treatment and the virus load was under the limit of detection. The baseline clinical characteristics of the enrolled patients were shown in Table 3. The protocol for the clinical trial conformed to the ethical guidelines of the Declaration of Helsinki and the enrolled populations were recruited with ethics committee approved by Xijing Hospital of The Fourth Military Medical University (Xi’an, China). Formal written informed consent was obtained from each patient.

**Table 3:** Baseline clinical characteristics of the enrolled patients

**Characteristics**	**Patients (n=42)**
Sex(m:f)	30:12
Age(year)	45.8±8.9
Albumin(37-55 g/L)	29.25±3.80
Total bilirubin(3.4-20.5 umol/L)	33.8±18.25
prothrombin time(11.0-15.0s)	19.13±2.98
ALT(0-40 IU/L)	50.83±27.12
CTP score	9.05±1.94
MELD score	14.04±4.89

Values are expressed as the mean±standard deviation, number or medians (range).

CTP, Child-Turcotte-Pugh; MELD, Model for End-Stage Liver Disease.

#### Follow-up

All patients were followed up for more than 1 year during this study. Clinical manifestation and physical examination were performed at 3-month intervals during the follow-up. At each visit, the concentrations of serum ALB, PTA, and CHE were measured to monitor the liver function of patients. CTP and MELD scores were calculated to assess the kinetics of the liver function of patients. During our follow-up, enrolled patients received regular anti-viral treatment and the virus load should be under the limit of detection. If virologic breakthrough or resistance occurred, “add on” or “switch to” another effective anti-viral drug would be adopted in according to guideline-recommend rescue measure. Venous blood samples were collected from all enrolled patients at each visit for the text of serum cytokines. The end of follow-up was December 2011. Endpoint was defined as the end of follow-up.

### Enzyme-linked immunosorbent assay (ELISA)

Serum levels of TNF-alpha, IFN-gamma, IL-12, IL-13, IL-4, TGF-beta1, IL-17, and IL-8 in enrolled populations were measured by ELISA kits (R&D Systems, Abingdon, UK) according to the manufacturer’s instruction. All the samples were assessed in duplicate. Plates were read using the Thermofisher (Massachusetts, America) microplate reader and cytokine concentration was calculated from the standard curve by the plate-reader software.

### Animal studies

#### Animals and treatments

Male C57BL/6 mice (10 to 12 weeks old, 20 to 28 g) were obtained from the animal center of the Fourth Military Medical University (Xi’an, China). They were provided food and water ad libitum and maintained on a 12-h light/dark cycle. All animal experiments were performed in accordance with guidelines from the Fourth Military Medical University Institutional Animal Care and Use Committee. Animals were assigned to groups randomly and we adopted a new procedure for inducing liver injury [33] with slight modification. In brief, liver injury was induced by intraperitoneal injection of 0.2 ml of a mixture of CCl_4_ and olive oil twice weekly for 6 weeks. The percentage of CCl_4_ in the olive oil (v/v) was increased gradually over time as follows: week 1, 13%; week 2, 16%; week 3, 20%; and weeks 4 to 6, 25%. Control mice received olive oil only.

#### Bone marrow stem cells preparation

C57BL/6 mice were killed by CO_2_ asphyxia and their limbs were removed for bone marrow isolation. The bone marrow was flushed from the femurs and tibias with serum-free RPMI-1640 (Gibco BRL, Carlsbad, CA) medium using 25 G needle, filtrated and centrifuged at 400 g for 5 min. For elimination of red blood cells, the cells were incubated in an RBC lysis solution (Gibco BRL) for 3 min.

### Examination of liver injury

Liver injury was determined by measuring serum ALB, as well as H&E and Sirius red staining. The liver fibrosis area was quantified with Sirius red staining as described previously [34]. Briefly, the red areas with Sirius red staining were assessed at 100 × magnification with a quantitative image analyzer. The mean value of five randomly selected areas per sample was used as the percentage of fibrotic areas.

### Measurement of IL-17 levels

The serum IL-17 levels were measured by ELISA using a Ready-SET-GO! Mouse IL-17A kit from eBioscience (San Diego, CA) according to the manufacturer’s instructions.

### RT-PCR analysis

Total RNA was extracted from 50 mg fresh or frozen liver tissues using RNA extraction kits from Takara Bio (Otsu, Japan) according to the manufacturer’s instructions. RNA (1 μg) was reverse-transcribed to cDNA at 37°C for 15 minutes and 85°C for 15 seconds using Superscript I kit (Takara Bio). Primers for alpha-SMA, collagen-1 and beta-actin were predesigned and validated by Takara. Primers were as follows: forward, 5′-AAGAGCATCCGACACTGCTGAC-3′, reward, 5′-AGCACAGCCTGAATAGCCACATAC-3′ (alpha-SMA); forward, 5′-GACATGTTCAGCTTTGTGGACCTA-3′, reward, 5′-GGGACCCTTAGGCCATTGTGTA-3′ (collagen-1); forward, 5′-CATCCGTAAAGACCTCTATGCCAAC-3′, reward, 5′-ATGGAGCCACCGATCCACA-3′ (beta-actin). beta-actin primer was used for internal control. RT-PCR was performed using Express SYBR Green (Takara). All reactions were performed in triplicate. Levels are expressed relative to matched control samples from the same time points. The specificity of the amplification products was confirmed by ethidium bromide-stained 1.5% agarose gels.

### The effect of IL-17 on the pathogenesis of liver injury induced by CCl_4_ administration in mice

Monoclonal rat anti-mouse IL-17 antibody (anti-IL-17 mAb), rat IgG2a isotype and rmIL-17 were purchased from R&D Systems (Abingdon, UK). On days −1, +1, +3, +5 and +7 after 6-week CCl_4_ administration, mice were injected anti-IL-17 mAb intraperitoneally (100 μg of anti-IL-17 mAb in 0.2 ml of sterile phosphate buffer solution (PBS)) (five total injections) in non-fasting condition. As a control, rat IgG2a was administered. RmIL-17 was intraperitoneally injected (1 μg of rmIL-17 in 0.2 ml of sterile PBS containing 0.5% BSA) on day −1 after 6-week CCl_4_ administration in nonfasting condition. As a control, vehicle was administered. Mice were sacrificed after 6 weeks of CCl_4_ administration.

### The role of IL-17 in the therapeutic effect of homogeneous BMSCs transplantation on CCl_4_-treated mice

**Figure 6:**
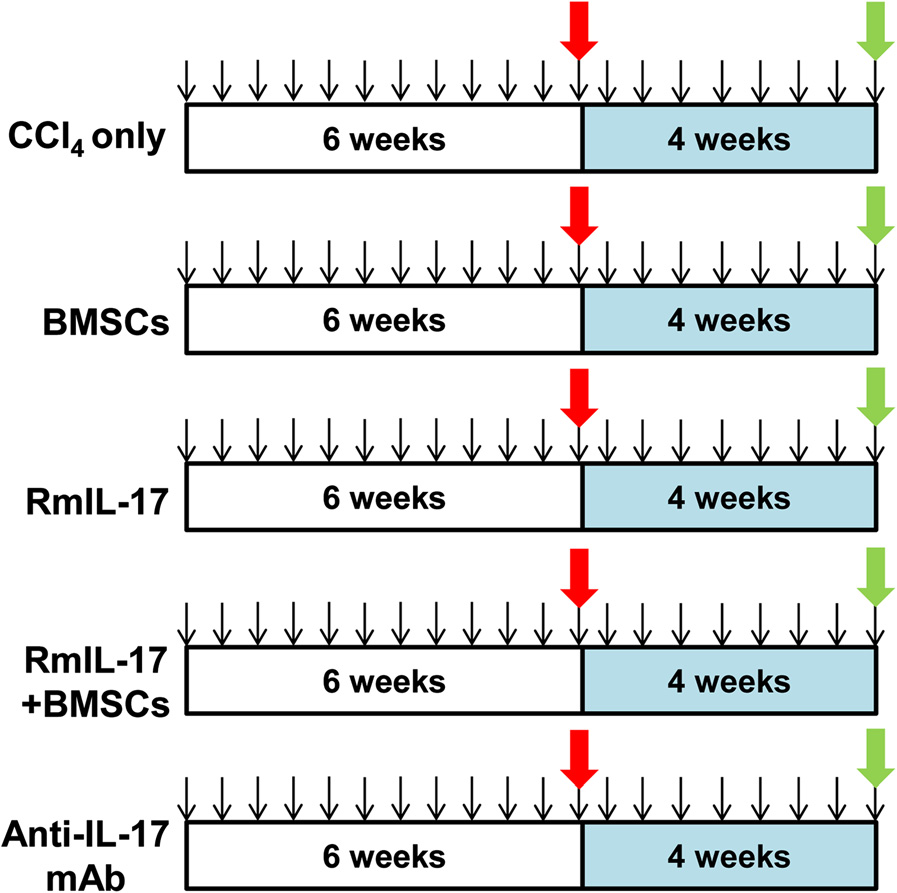
**The study design for the role of IL-17 in BMSCs-mediated amelioration of liver injury.** After 6 weeks of CCl_4_ administration to induced liver injury, the mice were randomly divided into 5 groups and then were given a tail vein injection of vehicle, a tail vein injection of BMSCs (5 × 10^6^ cells in 0.2 ml PBS), a intraperitoneal injection of rmIL-17, a intraperitoneal injection of rmIL-17 and a tail vein injection of BMSCs, and five intraperitoneal injections of anti-mouse IL-17 mAb, respectively (red arrowhead showed the time point). Then the mice continued to be given CCl_4_ injection during our study to maintain liver fibrotic status. Black arrowhead, the time of CCl_4_ administration; green arrowhead, sacrificed time.

As shown in Figure 6, after 6 weeks of CCl_4_ administration to induced liver injury, the mice were randomly divided into several groups. Mice in BMSCs group were given a tail vein injection of BMSCs (5 × 10^6^ BMSCs in 0.2 ml PBS), in rmIL-17 group a intraperitoneal injection of rmIL-17, in rmIL-17 + BMSCs group a intraperitoneal injection of rmIL-17 and a tail vein injection of BMSCs, and in anti-IL-17 mAb group five intraperitoneal injections of anti-IL-17 mAb. The control groups were given vehicle, or rat IgG2a injection. Then the mice continued to be given CCl_4_ injection during our study to maintain liver fibrotic status. One group mice were only given CCl_4_ injection during the whole study and served as control. Mice were sacrificed at the end of 10-week CCl_4_ administration.

### Statistical analysis

Comparison between groups was analyzed by the Student’s *t* test or ANOVA. The Spearman rank correlation test was used for correlation analysis. Data are reported as means ± standard deviation (SD). A *p* value lower than 0.05 was considered to be statistically significant. Statistical analysis was performed by GraphPad Prism 5.0 (GraphPad Software, Inc., La Jolla, CA) on a personal computer with Windows 7 operating system.

## Abbreviations

BMSCs: Bone marrow-derived stem cells; CCl4: Carbon tetrachloride; CTP: Child-Turcotte- Pugh; ALB: Albumin; PTA: Prothrombin activity; CHE: Cholinesterase; MELD: Model for End-Stage liver disease; ELISA: Enzyme-linked immunosorbent assay; RT-PCR: Real time-polymerase chain reaction; MSCs: Mesenchymal stem cells; SMA: Smooth muscle actin; H&E: Hematoxylin and eosin.

## Competing interests

The authors declare that they have no competing interest.

## Authors’ contributions

YS and YH were involved in study design, supervised the results of statistical analyses, edited the paper, and approved the final version. LZ and JC contributed to initiate the study, analyze the data, and draft the paper. XZ, LT, QL, and LC helped with data collection, data analysis, and paper revision. XW and ZH helped enroll and monitor patients, collect sample, and create figures for manuscript; MD provide study design and edit the manuscript. All authors read and approved the final manuscript.
